# Diagnostic Value of D-Dimer and INR in Patients Suspected to Have Prosthetic Valve Dysfunction

**DOI:** 10.21470/1678-9741-2021-0230

**Published:** 2022

**Authors:** Reza Heidari Moghadam, Nahid Salehi, Mohammed Rouzbahani, Parisa Janjani, Sousan Mahmoudi, Mohadeseh Izadpanah, Fatemeh Heydarpour, Ebrahim Shakiba

**Affiliations:** 1 Cardiovascular Research Center, Health Institute, Kermanshah University of Medical Sciences, Kermanshah, Iran.; 2 Medical Biology Research Center, Health Technology Institute, Kermanshah University of Medical Sciences, Kermanshah, Iran.; 3 Department of Clinical Biochemistry, Kermanshah University of Medical Sciences, Kermanshah, Iran.; 4 Behavioral Disease Research Center, Kermanshah University of Medical Sciences, Kermanshah, Iran.

**Keywords:** fibrin fragment D, Predictive Value of Tests, Sodium Citrate, Clergy, Diagnostic Tests, Routine, Anticoagulants, Biomarkers, Heart Valves

## Abstract

**Introduction:**

Prosthetic valve dysfunction is a potentially critical complication of heart valve replacement. An easy and quickly applicable diagnostic procedure is required for recognizing the prosthetic valve dysfunction. The purpose of this study was to prospectively define the diagnostic value of D-dimer and INR level in predicting prosthetic valve dysfunction.

**Methods:**

This cross-sectional study was performed in 70 patients suspected to have prosthetic valve dysfunction admitted to Imam Ali Hospital, affiliated with Kermanshah University of Medical Sciences (KUMS), Kermanshah Province, Iran. Cinefluoroscopy, as the gold standard diagnostic test, was used for the diagnosis of prosthetic valve dysfunction in enrolled patients. Two milliliters of blood from each patient were taken into a tube containing sodium citrate anticoagulant. To evaluate D-dimer, the cutoff value was set at 500 ng/ml. Also, to evaluate international normalized ratio (INR), the cutoff value was set at 2. Sensitivity, specificity, positive predictive value (PPV), negative predictive value (NPV), positive likelihood ratio (PLR), and negative likelihood ratio (NLR) of the serum markers were used to describe predictive properties.

**Results:**

Of 70 patients, 27 (38.6%) were male and 43 (61.4%) were female, and the mean age was 54.67±15.11 years (range, 18 to 80 years). Of 70 patients, 27 (38.6%) had prosthetic heart valve malfunction demonstrable by fluoroscopy, and 19 patients (27.1%) had D-dimer levels >500 ng/ml. Elevated D-dimer levels (>500 ng/ml) have been indicated to have sensitivity of 70.4%, and hence an NPV of 84.3%, specificity of 100%, PPV of 100%, NLR of 0.3, and the infinity value of PLR for predicting prosthetic valve dysfunction. There was a significant relationship between fluoroscopy and D-dimer test (P=0.001). A kappa coefficient value of 0.745 indicated a substantial agreement between D-dimer and fluoroscopy testing. Mixing test (combination of D-dimer and INR) showed to have 100% sensitivity, and hence a NPV of 69.8%, specificity of 69.8%, PPV of 51.8%, NLR of 1.41, and PLR of 1.44 for predicting prosthetic valve dysfunction.

**Conclusion:**

D-dimer with moderate sensitivity and high specificity is an ideal marker for the diagnosis of prosthetic valve dysfunction in suspected patients. Enhanced plasma D-dimer level is not by itself diagnostic of a prosthetic valve dysfunction but may alert physicians to refer the patient for more detailed examination, preferably by fluoroscopy. Mixing test with 100% sensitivity can apply as a rule-out test.

**Table:** Abbreviations, Acronyms & Symbols

IAH	= Imam Ali Hospital
INR	= International normalized ratio
KUMS	= Kermanshah University of Medical Sciences
NLR	= Negative likelihood ratio
NPV	= Negative predictive value
PLR	= Positive likelihood ratio
PPV	= Positive predictive value
SPSS	= Statistical Package for the Social Sciences
TTE	= Transthoracic echocardiography

## INTRODUCTION

Prosthetic valve dysfunction is a potentially critical complication of heart valve replacement^[1,2]^. An easy and quickly applicable diagnostic procedure is required for recognizing the prosthetic valve dysfunction. The recognition of prosthetic valve dysfunction is almost easy in patients who present with clinical symptoms of prosthetic valve dysfunction.

Transthoracic echocardiography (TTE) and cinefluoroscopy, as the most accurate and valid diagnostic tests, singly or in combination, are usually used for the diagnosis of prosthetic valve dysfunction in patients with clinical signs and symptoms of prosthetic valve dysfunction^[3]^. TEE and cinefluoroscopy may not be available in all centers or may not be sensitive enough to show the prosthetic valve dysfunction in asymptomatic patients.

D-dimer, as a degradation product of cross-linked fibrin, has been known to be a beneficial marker of endogenous coagulation activation and thrombosis^[4,5]^. High plasma D-dimer levels have been seen in patients with peripheral vascular disease, venous thromboembolism, prosthetic valve dysfunction, and acute ischemic stroke^[6-8]^. Increased D-dimer levels may reflect prosthetic valve dysfunction.

Therefore, physicians are still in demand of quick, easy, relatively noninvasive, and applicable diagnostic methods for diagnosing prosthetic valve dysfunction. D-dimer, a simple test, is widely accessible and applicable and adds no extra burden as part of routine prosthetic valve assessment. The purpose of this study was to prospectively define the diagnostic value of D-dimer level in predicting prosthetic valve dysfunction.

## METHODS

### Setting and Design

This cross-sectional study was conducted at Imam Ali Cardiovascular Hospital, affiliated with Kermanshah University of Medical Sciences (KUMS), Kermanshah Province, Iran. Imam Ali Hospital (IAH) is a long-term, comprehensive public health facility serving the central side of Kermanshah. This hospital serves Kurdish populations with any cardiovascular illness, regardless of disease severity and socioeconomic status.

Seventy consecutive patients who were suspected to have prosthetic valve dysfunction between January 1^st^, 2020 and December 30^th^ 2020, were enrolled. In our study, all valve prostheses were mechanical. Patients with diseases that increase D-dimer, such as deep vein thrombosis, disseminated intravascular coagulation, pulmonary embolism, aortic dissection, etc. were excluded from the study.

Our participants were referred to the hospital with symptoms such as sudden shortness of breath and reduced valve noise and underwent cinefluoroscopy. Cinefluoroscopy, as the gold standard diagnostic test, was used for the diagnosis of prosthetic valve dysfunction in enrolled patients. Prosthetic valve dysfunction is considered as decreasing of prosthetic valve leaflets motion, and closure and opening restriction. As already mentioned, we considered cinefluoroscopy as the gold standard for the diagnosis of dysfunction. Doppler echocardiography may report a false positive result because it indirectly measures gradians. For instance, valve gradian may increases in hyperdynamic cases like tachycardia, anemia, hyperthyroidism, etc., while cinefluoroscopy directly measures valve leaflets.

Two milliliters of blood from each patient were placed in a tube containing sodium citrate anticoagulant (Becton Dickinson and Company, Franklin Lakes, New Jersey, EUA). Platelet-poor plasma was created by centrifugation at 3,000 r/min for 10 minutes. D-Dimer Plus kit and Innovance D-Dimer kit were used with Sysmex CA-7000 and Sysmex CS-5100 automatic coagulation analyzers, respectively. To evaluate D-dimer, the cutoff value was set at 500 ng/ml. Also, to evaluate INR, the cutoff value was set at 2. The preparation and detection process was strictly based on reagent instructions.

### Definitions

Sensitivity is the possibility that a truly infected person will test positive. Specificity is the possibility that a truly uninfected person will test negative. Positive predictive value (PPV) is the possibility that those testing positive by the test are truly infected. Negative predictive value (NPV) is the possibility that those testing negative by the test are truly uninfected. Positive likelihood ratio (PLR) means how much to increase the probability of having a disease, given a positive test result. PLR is the possibility a person with the condition tests positive (a true positive)/possibility a person without the condition tests positive (a false positive). Negative likelihood ratio (NLR) means how much to decrease the probability of having a disease, given a negative test result. NLR is the possibility a person with the condition tests negative (a false negative)/possibility a person without the condition tests negative (a true negative).

### Statistical Methods

Categorical variables are expressed by their relative frequency and compared using the chi-square test or Fisher's exact test. Sensitivity, specificity, PPV, NPV, PLR and NLR of the serum markers were used to describe predictive properties. To assess the agreement between D-dimer, INR and mixing test with cinefluoroscopy, the kappa statistic was applied. Kappa coefficient is used for the assessment of agreement or reliability between two or more measurements. Kappa coefficient can be interpreted as follows: values ≤0 as indicating no agreement and 0.01-0.20 as none to slight, 0.21-0.40 as fair, 0.41-0.60 as moderate to good, 0.61-0.80 as substantial, and 0.81-1.00 as almost perfect agreement. Statistical analyzes were performed using SPSS 23.0 software. Values were considered statistically significant if *P*≤0.05.

### Ethics

The Research Ethics Committee at the Deputy of Research of KUMS approved the study protocol in January 2019 (IR.KUMS.REC.1398.940). In addition, the participants were given a participant information statement and signed a written consent form. Individual personal information was kept confidential.

## RESULTS

Table 1 shows baseline characteristics of patients. Of 70 patients, 27 (38.6 %) were male and 43 (61.4 %) were female, and the mean age was 54.67±5.11 years (range, 18 to 80). The types of prosthetic valve were aortic valve (32, 45.7%), mitral valve (29, 41.4%), tricuspid valve (1, 1.4%), and both aortic and mitral valves (simultaneously) (8, 11.4%).

**Table 1:** Baseline characteristics of patients.

Characteristic	Category	Total (N=70)	Prosthetic valve dysfunction (N=27)	No prosthetic valve dysfunction (N=43)	*P*-value
Age	-	54.67±15.11	51.45±13.89	52.67±14.18	0.466
Gender	Male	27 (38.6)	11 (40.7)	16 (59.3)	0.768
	Female	43 (61.4)	16 (37.2)	27 (62.8)	
Prosthetic valve	Aortic valve	32 (45.7)	13 (40.6)	19 (59.4)	0.722
	Mitral valve	29 (41.4)	10 (34.5)	19 (65.5)	
	Tricuspid valve	1 (4.4)	0 (0)	1 (100.0)	
	Both aortic and mitral valves	8 (11.4)	4 (50.0)	4 (50.0)	
Hypertension	Yes	25 (35.7)	9 (36.0)	16 (64.0)	0.741
	No	45 (64.3)	18 (40.0)	27 (60.0)	
Diabetes mellitus	Yes	32 (45.7)	11(43.0)	21 (57.0)	0.508
	No	38 (54.3)	16 (42.1)	22 (57.9)	
Dyslipidemia	Yes	18 (25.7)	7 (38.9)	11 (61.1)	0.974
	No	52 (74.3)	20 (38.5)	32 (61.5)	

Of 70 patients, 27 (38.6%) had prosthetic valve dysfunction demonstrable by fluoroscopy, which 16 (59.3%) were female and 11 (40.7%) were male (*P*=0.768).

Based on fluoroscopy findings, 13 patients (48.1%) had aortic valve dysfunction, 10 patients (37%) had mitral valve dysfunction, and 4 patients (14.8%) had aortic and mitral valve dysfunction simultaneously (*P*=0.722).

Of 70 patients, 19 (27.1%) had D-dimer levels >500 ng/ml, of which 13 (68.4%) were female and 6 (31.6%) were male (*P*=0.463). Of 19 patients with D-dimer levels >500 ng/ml, 6 (31.5%) had aortic valve prosthesis, 10 (52.6%) had a mitral valve prosthesis, and 3 (15.7%) had aortic and mitral valve prosthesis simultaneously (*P*=0.434).

Of 70 patients, 35 (50.0%) had INR levels <2, of which 21 (60.0%) were female and 14 (40.0%) were male (*P*=0.806). Of the 35 patients with INR levels <2, 18 (51.4%) had aortic valve prosthesis, 12 (34.2%) had mitral valve prosthesis, 1 (1.4%) had tricuspid valve prosthesis, and 4 (11.4%) had aortic and mitral valve prosthesis simultaneously (*P*=0.500).

Moreover, 14 patients (20.0%) had D-dimer levels >500 ng/ml and INR levels <2 (mixing test).

Based on the Fisher’s exact test, there was a significant relationship between fluoroscopy and the D-dimer test (*P*=0.001). A kappa coefficient value of 0.745 indicated a substantial agreement between D-dimer and fluoroscopy testing. Based on the chi-square test, there was a significant relationship between fluoroscopy and the INR test (*P*=0.001). A kappa coefficient value of 0.486 indicated a moderate to good agreement between INR and fluoroscopy testing. Based on the chi-square test, there was a significant relationship between fluoroscopy and the mixing test (combination of D-dimer and INR) (*P*=0.001). A kappa coefficient value of 0.216 indicated a fair agreement between mixing test and fluoroscopy testing (Table 2).

**Table 2:** Relationship between fluoroscopy and biomarkers.

Characteristic	Positive fluoroscopy	Negative fluoroscopy	Kappa coefficient	*P*-value
**D-dimer ≥500**	8 (29.6)	43 (100)	0.745	0.001
**D-dimer >500**	19 (70.4)	0 (0)		
**INR ≥2**	5 (18.5)	30 (69.8)	0.486	0.001
**INR< 2**	22 (81.5)	13 (30.2)		
**Mixing test (D-dimer >500 & INR <2)**	14 (51.9)	13 (30.2)	0.216	0.001
**Mixing test (D-dimer ≤500 & INR ≥2)**	13 (48.1)	30 (69.8)		

Quantitative determination of D-dimer levels has been demonstrated to be a very useful tool for predicting prosthetic valve dysfunction. Elevated D-dimer levels (>500 ng/ml) have been indicated to have sensitivity of 70.4%, and hence NPV of 84.3%, specificity of 100%, PPV of 100%, NLR of 0.3, and the infinity value of PLR for predicting prosthetic valve dysfunction. The sensitivity, specificity, PPV, NPV, PLR and NLR were 81.5%, 69.8%, 62.8%, 85.7%, 2.70, and 0.27, respectively, for the INR. When evaluating the potential combination of these serum markers, we found that the combination of D-dimer and INR showed to have sensitivity of 100%, and hence a NPV of 69.8%, specificity of 69.8%, PPV of 51.8%, NLR of 1.41, and PLR of 1.44 for predicting prosthetic valve dysfunction (Table 3).

**Table 3:** Sensitivity, specificity, PPV, NPV, PLR and NLR of D-dimer, INR and D-dimer + INR.

Biomarker	Sensitivity	Specificity	PPV	NPV	PLR	NLR
**D-dimer**	70.4	100	100	84.3	∞	0.3
**INR**	81.5	69.8	62.8	85.7	2.70	0.27
**D-dimer + INR**	100	69.8	51.8	69.8	1.44	1.41

INR=international normalized ratio; NLR=negative likelihood ratio; NPV=negative predictive value; PLR=positive likelihood ratio; PPV=positive predictive value

## DISCUSSION

Mechanical valve prostheses have the advantage of longevity but carry a risk of obstruction (by clot or thrombosis) followed by prosthetic valve dysfunction. Malfunctioning of the prosthetic valve is one of the most dangerous and deadly complications in patients with valvular replacement. This complication often occurs due to inadequate anticoagulant therapy. Therefore, it needs quick diagnosis and timely treatment^[9]^.

TTE and cinefluoroscopy, as the main diagnostic procedures, are used alone or in combination for diagnosis of the prosthetic valve dysfunction^[10,11]^. However, TTE and cinefluoroscopy may not be available in all centers. D-dimer, INR and their combination, as beneficial markers of endogenous coagulation activation and thrombosis, may reflect prosthetic valve dysfunction^[12]^.

We examined 70 patients who were suspected to have prosthetic valve dysfunction. In our study, the prevalence of prosthetic valve dysfunction was 38.6% (27 patients) by fluoroscopy, with the highest involvement in the aortic valve (48%) and mitral valve (37%), respectively. Previous studies have found that valve thrombosis, as a potentially dangerous complication, is more common in aortic and mitral valves based on clinical examinations^[13,14]^.

The present study found that D-dimer, INR and their combination were useful in predicting prosthetic valve dysfunction. Among them, D-dimer may have the potential for an earlier prediction of prosthetic valve dysfunction. Elevated D-dimer levels (D-dimer >500 ng/ml) showed sensitivity of 70.4%, *i.e.*, NPV of 84.3%, specificity of 100%, PPV of 100%, NLR of 0.3, and the infinity value of PLR for predicting prosthetic valve dysfunction. This means there is a relatively high false-negative rate (29.6%) for diagnostic accuracy of D-dimer but the false-positive rate was 0%. This means that a negative result will need further investigation with TTE and cinefluoroscopy while a positive result is most probably suffered from malfunctioning of the prosthetic valve. Furthermore, a kappa coefficient value of 0.745 indicated substantial agreement between D-dimer and fluoroscopy testing.

Determination of plasma D-dimer levels seems to be a useful tool in early predicting prosthetic valve dysfunction. It was observed that D-dimer levels >500 ng/ml predicted the presence of a prosthetic valve dysfunction with high specificity and moderate sensitivity. In accordance with our results, Nazli et al.^[15]^ reported that high levels of plasma D-dimer (>445 µg/L) predicted the presence of a prosthetic valve thrombus with high specificity and moderate sensitivity. The reason for the moderate sensitivity may be due to that D-dimer levels were affected by the presence of the prosthetic valve, as well as the level of coagulation activity.

In the literature, there are divergent opinions about sensitivity and specificity of D-dimer. Castro et al.^[16]^ reported that sensitivity and specificity of plasma D-dimer in the diagnosis of pulmonary embolism are 92 and 71%, respectively. Jiang et al.^[17]^ reported that the D-dimer level could predict the development of deep vein thrombosis, with the highest sensitivity of 71.4% and specificity of 81.7%, in 2015. Dong et al.^[18]^ in 2017 demonstrated that the sensitivity and specificity of D-dimer were 68.8 and 60.9% in predicting aortic dissection. Suzuki et al.^[19]^, in a study involving 220 patients with clinical suspicion of aortic dissection, reported a sensitivity of 97%, specificity of 47%, and NPV of 97.6% for D-dimer. A meta-analysis with a pooled population of 734 patients reported a sensitivity of 96% and a specificity of 56%, with a NPV of 96% for D-dimer in diagnosing aortic dissection^[20]^.

Likelihood ratios are important reference indicators for physicians. They provide information about the probability that a patient with a positive or negative test result will have a prosthetic valve dysfunction. According to the result of our study, PLR of infinity for D-dimer implies that a patient with prosthetic valve dysfunction is infinity times more likely to have a positive test than a healthy person. Likewise, the NLR is 0.3 for a negative test result.

It has been found that INR levels are a prominent predictor of D-dimer levels and a significant predictor of thrombus formation in prosthetic valves. Georgiadis et al.^[21]^ and Giansante et al.[^22^] have reported that D-dimer levels were higher in patients with prosthetic valves who had an INR <2.0, comparing those who had an INR >2.0. An INR <2.0 showed 81.5% sensitivity, *i.e.*, an NPV of 85.7%, specificity of 69.8%, PPV of 62.8, NLR of 0.27, and PLR of 2.70 for predicting heart valve dysfunction. There is a relatively high false-positive rate (30.2%) for diagnostic accuracy of INR, but the false-negative rate was 14.3%. Moreover, a kappa coefficient value of 0.486 indicated a moderate to good agreement between INR and fluoroscopy testing.

Our results demonstrated that the combination of D-dimer and INR showed to have sensitivity of 100%, and hence a NPV of 69.8%, specificity of 69.8%, PPV of 51.8%, NLR of 1.41, and PLR of 1.44 for predicting prosthetic valve dysfunction. There is a relatively high false-positive rate (30.2%) for diagnostic accuracy of mixing test but the false-negative rate was 0%. Actually, a negative result was probably not caused by a malfunctioning prosthetic valve. This means that the mixing test with 100% sensitivity can be applied as a rule-out test. A kappa coefficient value of 0.216 indicated a fair agreement between mixing test and fluoroscopy testing.

### Limitations of the Study

Several limitations of this study can be addressed. First, the number of patients who developed prosthetic valve dysfunction was insufficient. Second, the administration of various drugs after a valve replacement for patients probably also influenced the D-dimer levels. However, it was difficult to avoid this effect. Third, our single-hospital experience may not be generalized to the broader community.

## CONCLUSION

Overall, our analyses enhanced the database of knowledge for the diagnostic value of D-dimer for predicting prosthetic valve dysfunction. As our results show, D-dimer with a moderate sensitivity and high specificity is an ideal marker for the diagnosis of prosthetic valve dysfunction in suspected patients. Enhanced plasma D-dimer level is not by itself diagnostic of prosthetic valve dysfunction but may alert physicians to refer the patient for more detailed examination, preferably by TEE and cinefluoroscopy. Current data also suggest that the mixing test with 100% sensitivity can apply as a rule-out test. Plasma D-dimer and INR assay is a mixing method now easily available as an emergency test. Future clinical studies with different assays are required to support these findings and to evaluate the probability of incorporating the D-dimer assay with other biomarkers. Moreover, further experimental research would be required in a larger number of patients with prosthetic valve dysfunction from several hospitals. A future survey for the use of the D-dimer assay can be applied in the selection of the best treatment for secondary prevention.
